# Professional Ethics

**Published:** 1906-02-15

**Authors:** T. R. Buttrick


### PROFESSIONAL ETHICS.

BY T. R. BUTTRICK, D.D.S.

Read before the Detroit Dental Society, December 14, 1905.

The question of ethics in the profession is one of right conduct, it includes ones duty to his patients, his professional confrers, and the public expressions of the profession in its social gatherings. In order that I might put myself in harmony with the expressed sentiment of the profession, I, for the first time, read over the printed code of ethics as adopted by the American Dental Association in 1866, and I commend it to every one as a guiding principle on this subject.

In the main we can agree with this code, although there may be some things we should slightly alter. It seems to me that the gist of it could be put into two sentences, already familiar to everyone. First, Do unto others as you would have others do unto you. Second, Put away commercialism and strive for professionalism in dentistry. This means that we should each do all that is practicable to put our calling on a stricly professional basis rather than a commercial one.

In discussing the subject I shall strive to do so from the judicial and impersonal view-point, and with my mind unbiased by any prejudices of a personal nature. I shall use the first person singular pronoun as the easier grammatical construction and not from any egotism on my part, for some of the statements I shall make, will hit myself as hard as any one else. It is far easier to criticise than to perform.

The nearer we all come to keeping the golden rule, the less we shall be hampered by the code of ethics.

As a general proposition I should say that the first duty of a dentist is to his patient, who confidently places himself in his care; and the second is perhaps to his fellow practitioners.

For the patient, advise no operation that you would not wish performed on yourself if your places were actually reversed, is a good rule. How often we encounter operations that seemingly were unnecessary. Here we must be charitable, even should we be convinced that the fee received was the controlling motive rather than the best good to the patient. A dentist told me recently that a patient came to him with a live bicuspid tooth having only a small mesial cavity, which another dentist had advised should be cut off and replaced with a Logan crown. A patient came to me recently for a partial upper plate, having only the six front teeth left in the upper jaw. Some dentist had placed three bridges in the lower jaw one between the cuspids, and one on each side from the third molars forward. The anterial bridge was in fair occlusal relations with the upper natural teeth, but the two bridges on the sides were so long that they almost occluded with the upper gums, rendering it impracticable to place a plate in the upper jaw without opening the bite of the front teeth to an extent that was not admissable. Now I contend that the dentist who advocated the Logan crown in the first case and the man who made these bridges were both knaves, although they pass as ethical gentlemen. They are really doing more injury to true professionalism than the advertising charlatans, who make no pretension except to give mercenary and gullible people something for nothing.

One sees so much execrably bad dentistry in the mouth of people that he can't help but think that the dentist who did it must bave been called to be a plumber or to have kept his eyes upon the patients pocket-book, rather than on the operation he was performing. I contend that when a patient comes to me and puts himself in my care, that it is my duty to put his entire mouth in as good a condition at it is practicable for me to do, and should I find defective work that is likely to interfere with the normal functions, or to cause injury to the patient, it is my duty to correct the same as far as it is possible to do so. By this statement I do not mean to infer that I would change everything that is not absolutely perfect, but that everything which may be detrimental to the best interests of the patient should be made right. Should I fail to do this, I should feel that I had not done for the patient that which I should expect him to have done for me, were our positions reversed.

I believe in being charitable with the work of my confrers, but I see so much work that is atrociously bad, that I can't but feel that anyone with average skill should have at least made an effort to improve the deplorable conditions. How often do we find fillings bulging out of the cavity walls, impinging on the gums and producing the most unsanitary results; on failing altogether to reproduce normal contour, both of the occlusal and proximal surfaces. Or crowns so inartistically made that they look—as Dr. Moore used to tell us at college—"like a tin can on a hitching post," with no attempt at fitting the root or restoring occlusion or proximal contact; or bridges whose offenses are too numerous to mention.

Then there is the question of extracting teeth unnecessarily. In this matter as in others the proverb that "dead men tell no tales" applies most readily to avoid adverse criticism. We must here use great caution as we cannot always say what should have been proper treatment, as we cannot know all the facts. As a broad principle I should say that no tooth should ever be extracted that can by any means be saved and made useful. This statement would, of course, be modified by some special circumstances or condition based on an honest opinion of what would be best for the patient in that particular case. I have seen so many mouths utterly ruined by ruthless extractions that I feel very strongly that it is not ethical to extract any tooth that may be made serviceable. I have several times absolutely declined to extract teeth that were diseased, but which I believed I could cure and make them useful servants for my patients, and thereby lost the patient, but I have always felt that I preserved my own self respect and have gained a better professional attitude by so doing. I feel that it is as reprehensible to amputate a sore finger as to break unnecessarily in to a complete denture of teeth that can be treated and saved.

A dentist once told me that he only extracted such teeth as he could extract with his fingers. This was doubtless an exaggerated statement, but expresses an attitude of mind that is commendable. He propably extracted supernumerary teeth and impacted third molars with the forceps.

The other day an old gentleman came to me with a very sore filled tooth, with a history of its having been devitalized by another dentist—an ethical one by the way—after he had blundered into the live pulp while preparing the cavity for the filling. After vainly endeavoring to subdue the irritation by counter irritants, I was compelled to open into the pulp canal. It was a lower first molar and the filling had been securely anchored into the pulp chamber. I found a suspicion of gutta percha in the distal canal, but the mesial canal had never been opened. The canals were difficult to locate, but I finally succeeded in opening into the buccal division and a free flow of pus resulted. Now I was compelled to believe the dentist either imcompetent or a rascal, and I do not think in either case he should be entitled to much consideration as a professional confrere. I do not believe it right or even good policy to "knock" the other dentist in such cases, as no good can result and perhaps a bad impression will follow. We should enlighten our patients as to actual conditions, but we can and should exhonorate our confrere, as we cannot always know the circumstances. I have on more than one occasion encountered operations in mouths that I have classified as exceedingly deficient, and then when I have undertaken operations in the same mouth, I find circumstances to contend with that in a large measure account for the quality of the work previously done. The patient may be irritable to such an extent that it is utterly impracticable to do more than to temporize. Then we must be charitable as the best of us with the highest intentions will sometimes fall far short of doing creditable operations. "Let him that is without sin cast the first stone." As a matter of fact an occasional failure can do us little harm and may be the making of us. It may keep us from getting "chesty," and if we are of the right kind of stuff, we shall strive harder in the future to prevent its recurrence. It may be hard on the patient, but he usually does'nt know how sadly we have failed, for people of intelligence will usually be satisfied with any thing, however abominable, that gives them no special inconvenience. For instance, I have a cap crown on one of my molars that is atrociously ill fitted, and yet before I entered the dental profession I supposed it was the acme of dental art.

On the contrary a greater obligation rests on us ethically to commend operations that we encounter that are what they should be, this for the good of our associates and the profession, but it usually redounds to our own credit. I remember once speaking favorably of two small gold fillings in the mouth of a patient, and the patient said: I know those fillings are good, and should you have said anything against them, I should never have come to you again. As a result this lady has become a loyal patron and sends me all her family and friends.

Advertising for business is considered a professional sin, but it occurs to me that it is the commercialism that prompts it that is to be blamed. Let us be sensible and honest with ourselves. When we were students in college and wanted patients to practice on, we did not consider that any breach of ethics occured when the management advertised in the public prints for patients to supply our own needs. When we advertise cheaper dentistry than the general profession can afford to give, do we not take an unfair advantage of some one, whether we are conducting a dental college or a private practice? I now number among my valued patrons some patinets for whom I worked while a student in the college, attracted to the college clinic by the advertising above referred to. I am, however, convinced that the most desirable class of patients will not come to one as a result of any public advertising. I believe it would be possible to conduct an advertising business on a fair and legitimate basis so far as I am personally concerned, but I don't think anyone could do this and be considered professionally ethical, since the bulk of the profession does not resort to this means for securing business. The worst feature of the usual advertising dentist is that he does not perform his promises; he renders poor services and collects larger fees than his service warrant. There are of course exceptions. I know an advertiser in this city who does excellent work and charges reasonable fees. I respect such a man really more than I can the so-called ethical man who pretends and does not perform the services the patients ought to have and expect.

We all advertise more or less, in some way or other, some of these ways are legitimate and honorable, while others are reprehensible in the extreme and should bring a blush of shame to the perpetrators. I have far more respect for the out and out commercial dentist than I have for the so-called ethical practitioner, who goes about like a wolf in sheep's clothing, preying upon his confiding patrons, by performing unnecessary operations, and charging them exorbitant fees. I believe the advertiser does far less harm to the profession than such a man who supposedly stands before the public as an ethical representative of a noble profession which he debauches by practicing it as a charlatan.

There are legitimate ways of securing patrons on an honorable basis, one of the best means of putting yourself favorably before your patrons is in the matter of your office surroundings. Select your furnishings and equipment with the best possible taste, and keep your office as well as your person always in the most acceptable manner. Do not think you can afford other things before these. Make it your rule to render every service as complete as you possibly can regardless of the fee you are to receive for it. A well pleased patient is the best advertising medium you can have. By serving every patient faithfully and acceptably, we soon accumulate a wealth of advertising agents that constitute a most valuable business asset of permanence. We should do our own laboratory work if possible. I should not, under any circumstances, put my work into the hands of the concerns which make such extravagant claims as some of them have been doing the past year in our city.

A friend of mine sent a gold plate to one of these laboratories and they made a fearful piece of work of it, and what is worse, refused to make any reparation for a piece of work that would disgrace the clumsiest freshman student. In reply to one of their letters I wrote them that if their treatment of my friend was a sample of their business methods, and the plate in question was a sample of their skill, I had no use for their laboratory and they could take my name from their mailing list.

As professional men we ought to guard against the endorsement of business schemes that we know little about, especially proprietary remedies that innocent people would suppose we were competent judges of their merits. And I very much doubt the propriety of a dentist having a financial interest in tooth toilet articles, for instance, which he is thereby compelled to recommend to his patients. We should always be in a position to change our ideas as the value of any remedy without commercial restraint. I have been repeatedly solicited by a company making mouth toilet articles of great value so far as I can judge, to buy some of the stock and thereby have an interest in the profits of the business, which of course is to be increased because of my recommending its products to my patients. I have declined because of the ethical relations involved, and also because I think I can use my own money to better advantage.

There is a business side to our work which requires close attention, but it is not of the commercial character that I have been discussing. It is in many cases sadly neglected by the dentist. It is the keeping of his own accounts in a systematic manner. I believe thoroughly in the keeping of accurate records with the time spent on each operation, and by this means we have a correct basis for our fees. A minimum fee for time spent and cost of each operation as a basis, will give us an opportunity to modify the fee in individual cases. The fixing of fees is a matter of judgment and education. Good fees should mean good services. We are justified in this because of our long and expensive training, and on a time basis we should demand dignified fees that are commensurate. As a business policy it is good practice to inform patients when expensive operations are to be made, especially if they are new patients. It is very unpleasant to have a business misunderstanding with our patients because of a supposed overcharge. It is bad policy to discount a bill when once made, as it is evident that you realize you have made an overcharge, and this makes a bad impression, it also gives you the reputation of a rebater, which is not at all good. People who won't pay their bills to a dentist are the worst kind of dead beats, as they generate a class of dead beats that are a nuisance.

I cannot too strongly urge the constant and prompt attendance in society meetings, and participation in the proceedings. We don't realize how much good we can get out of a meeting until we take a personal part in it.

I want to say a word about the attitude of our colleges on this question of ethics, and what I shall say applies no more to my own college than it does to any other. "It is only an ill bird that fouls it's own nest." It has always seemed to me that our colleges are directly responsible for some of the crying evils of our profession. In the class in which I graduated there were over fifty students not one of them was plucked, they certainly, to my knowledge, were not all sufficiently qualified to begin practice. I perhaps should not complain, as they even let me through. But I should not be far from making a correct statement should I say that some of these men scarcely ever looked into their books, as the majority never owned many. If our profession is to become learned and honored, the colleges as the gateways must do the screening. In the first place if they set a standard of admission it should be religiously enforced, and should not be made a farce by allowing entrance conditions to be removed by a species of kindergarten work for a small fee paid to some instructor. The passing of time should be no guarantee that a student is qualified regardless of failures to attend to his work and accomplish results in the class room and laboratories. The aging process may be good in some things but it requires actual work to make a dentist who is worthy a diploma, which permits him to practice on an unsuspecting public. Students should be examined at stated intervals and made to show progress or be excluded from attendance at the college. A student should not be carried through several years and made to pay in all his coin, except the graduation fee, only to be turned down then. It ought to be possible to determine at the end of the first year whether a student will make a dentist or not, and he should be given this information for his own good as well as for the general professional good. It seems to me that a college conducted on any other basis must be run for the student's fees only, and therefore can be but little better than a diploma mill which will issue its diplomas for so much money, regardless of a curriculum.

It would seem to the disinterested person that the demand for a four-year course had no other motive than to secure another fee from students, and the colleges quickly retracted when they found that the attendance was to be cut to less than half of the former number.

Now if the colleges do not conduct their affairs on a high ethical basis, should we be surprised to find that a large number of college graduates are found practicing unethically? Students should be taught ethics not only from the rostrum, but in every business transaction which passes through the college. Educate the young men in college to be honest and industrious and they will not aim merely to get their degrees. This subject need to be taught theoretically and also be worked out in an exemplary manner before the students. It is one subject which should form an important item of every dental college curriculum. There will be enough young graduates going wrong at best, but it would certainly be a great help to the student to be put face to face with his obligation to society and his profession at the very beginning of the course.

DISCUSSION.

DR. THOMPSON: It seems to me rather a hard matter to write a paper upon the question of professional ethics without almost giving a theological discourse. Unless we enter into it from personal observation and throw a great deal of our personality into it, as Dr. Butterick has in his paper, it is pretty hard work to bring out the points you so much desire.

Dr. Buttrick did not give us any definition of the word "Ethics." He says he did not hear it all while he was in college. I can say that I heard it once, as I wrote a thesis on professional ethics. Webster defines ethics as the science of human duty; deteimining whether true or false, etc. Another authority says: "Ethics is the science of the laws which govern our actions as moral agents."

Dr. Buttrick spoke of the Golden Rule. I believe it is generally conceded that the sermon on the mount given by Christ is the embodiment of all true living, and if every other teaching in the Scripture were removed, there is enough in these three chapters to give us the plan for right living and doing justice to our neighbor. The first verse in the chapter reads: "Judge not lest ye be judged." The second verse: "With what judgment ye meet it shall be measured to you again." The third verse: "Why beholdest thou the mote that is in thy brother's eye and considereth not the beam that is in thine own eye; or, why sayest thou to thy brother: let me pull out the mote that is in thine eye, and beholdest not the beam that is in thine own eye."

A paper was read before some gentlemen at Moline, Ills., last spring, which was given in the *Review*. That man took this position: If we drive fast horses and ride in automobiles we were advertising; if we belonged to a church we advertised and we were all grafters, so to speak. If we carried out his theories entirely we would not be anything; we would not have any personality. I think if a man is in business and wants to have a good horse or ride in his automobile, that is his business and nobody elses. I think that when a man goes out of town, if he wants to put it in the paper that Dr. so and so is spending a few days in such a place, that is his business. There are lots of ways of being ethical and advertising, besides advertising in the newspapers. Some one handed me this the other day: Dr. so and so down the street says that Dr. Thompson goes to church, while I advertise in the newspapers. I know I go to church. Now is that advertising or doing our duty as we see it? If I have to quit going to church to be ethical, I am not going to be ethical.

Another feature of our attitude towards each other sometimes is due to the fact that we do not stop to realize what the other fellow's position is. When I first came to Detroit some of the persons who are now present I do not consider were very friendly to me, and I am frank to say that I said things that would not have appeared pretty in print about them, but as we get acquainted and the outer scale falls off, we find out that these very people are ready with the glad hand to do for us if we only do justice by ourselves.

About two years ago I wrote to Dr. J. Foster Flagg, and I was thinking then of getting up a paper on the subject of "Would Commercialism and Ethics Ever Coalesce?" The answer I received I wish that we could all read it. The substance of his letter was that commercialism and ethics were no nearer together at this time than they had been; one was the everlasting joy of giving and the other the everlasting thought of getting. After Dr. Flagg went into the manufacture of amalgams he resigned from every society to which he belonged. He said that he could not be ethical and a commercial man at the same time.

Another part of the sermon I spoke of, and this I think applies to the college persons just as much as to anyone else, "By their fruits ye shall know them."

DR. S. STRAITH: I think we may each congratulate himself that the other fellow was called upon to write upon the subject which was alloted to Dr. Buttrick, and then we deserve another congratulation that the choice fell upon one who is able and fearless enough to give us the good paper we are privileged to hear and to discuss. To some of us it is not an easy matter to write upon any subject, but to be asked to tell how little we *know* and how much we *think* about *Professional Ethics* would be enough to bring out all the elements of our nature that help to spell the words *no* and I *can't*.

While we compliment the essayist upon his paper, we also know he would think us insincere if we did not differ from him on some point.

The essayist starts off with the advice to "put away commercialism and strive for professionalism." This *sounds* very well and looks very well on paper, but when I see any dentist who has attained to that condition of mind and practice I shall at once begin to look for his wings, and should I find them I shall venture to tell him "the earth is not his dwelling place." Had I not chanced to read the article of B. T. Ledyard Smith on "Working for Money" in the October number of the *Items of Interest*, I might attempt to give some thoughts upon the subject; but Dr. Smith has expressed himself in such language that any attempt of mine would seem very tame to any who had read his article. If the essayist read Dr. Smith's article before writing his paper, then I commend him the more heartily for the fearless attitued he takes; but if he did not, then I recommend Dr. Smith's article to him as a little missionary tract to convert him from the errors of his ways. Dr. Butterick's plea for charity in our criticism upon others will be heartily concurred in by us all. It is so easy for us to find imperfections, and if we were half as anxious to locate and profit by our own mistakes as we are to see those of others, we would be much happier ourselves and the product of our labors would tend more to perfection.

There is probably no one amongst us who has a better opportunity to view the work of the "other fellow," than myself; but I have trained myself to not see any of the other teeth than the ones upon which I am to operate. There are, however, very often pieces of work in a patients mouth so noticeably good or so conspicuously bad, that they can scarcely escape my notice. When ever it is of the former I usually compliment the operator whoever it may be; when it is of the latter, I say nothing. If I am asked to pass judgment upon another dentist's poor work, I strive to evade the question, unless I know it to have been done by some one whose very life and continual practice does not entitle him to fraternal protection, then I feel that in exposing the operator, I have done a duty I owe both to my patient and to my fellow practitioner. The essayist has said that in criticising the work of another we should remember that circumstances very often materially alter cases. We are not all built alike. I am unable to attract all classes of people, and it is quite likely that some who feel quite at home with me would feel ill at ease in another's chair. It is only quite natural that if some one of you very amiable patients should by mistake, or otherwise, come into contact with my nature which is so unlike yours, it would be a disappointment to me if I got results from my labors as satisfactory to myself as if I had been working for one of my own patients between whom and myself there was more of an affinity. Now then, I claim that whenever this patient wakes up from his dream and goes back to you, where every thing just suits him, it is quite unjust of you to criticise too harshly that piece of work of mine, which in all probability does not suit me any better than it does you, but the conditions or circumstances influencing this case may be only one of many which may have helped to influence the result of this or some other operation. Then there are very few of us who are all around good men. As long ago when you attended school you remember there were some who excelled in spelling, others who have scarcely ever been able to spell their own name and be sure of it, others who were good grammarians and others who from positive *apparent* inability to comprehend the study, would play hookey from school to get away from that study. In dentistry it is difficult to find a man who is equally proficient in putting in a gold filling, making a crown or a continuous gum plate. A dentist is in a general practice and he has the time and ambition to put in a bridge. He may be attempting one of his first jobs in that kind of work, the finished product may represent his best conscientious effort, and his untutored eye may not detect the imperfections that your experienced eye will see at a glance.

Are you at liberty to berate his work and put him down as a base deceiver and a charlatan because his work does not compare with yours? This may be one of the hard words for him to spell, but he means to persevere and some day he may turn out work in this line that will compare very favorably with your present efforts and possibly he may be able to do some little thing in dentistry *now* that would put to shame your best effort in the same line. You may say that if he cannot do a better job than that he had better turn over the work to some one else. He may not be in favor of specialists, but of course if it were a case of the extraction of a tooth I would be inclined to agree with you. A dentist's liking for or dislike to certain lines of work has much to do with his fees for such work, notwithstanding the commercial spirit should be eliminated we are told. If I enjoy and take pride in putting in gold fillings I shall turn out work people will appreciate, and for which they will pay me good fees, probably much better fees than you can get for your gold fillings, because you are more facinated with your crown work and in turn will get better fees for that work than I can get for mine in the same line. Now, I contend that each of us, should we come in contact with the other fellow's patient, ought to uphold him to the patient in charging a good fee for the work in which he has bent his efforts to excel. That filling put in by the other dentist may not compare at all with the one you could have put in in the same place, but he did his best and then charged only a fraction of the price you would have received for your filling. I believe there are many of you who would rather any time put on a Richmond crown, a jacket crown or a bridge, than to extract almost any lower third molar; yet when I extracted the lower wisdom teeth and one upper wisdom tooth for a patient who is abundently able to pay *any* fee for her work, she was allowed, by her dentist to think that my fee, which was less than one-third of the fee for a jacket crown, was an exorbitant one and I an extortionate charger. The contention was that the teeth were not difficult of extraction and gave me no trouble at all. I trust it may not be considered altogether egotistic if I make the comparison that it is barely possible that that jacket crown which you put on so easily and so nicely, I might not have been able to put on at all, to say nothing of the pain and inconvenience I might have caused the patient. The essayist refers to the needless extraction of teeth which expression will bring a hearty response from all of us. Very often people come to me for the extraction of teeth without the advice of a dentist. If such a person has a regular dentist, I invariably refer him to his dentist, if I have the least idea that the tooth can be saved. If he has no dentist and can be persuaded to go to one, he is so advised, and if he asks me to refer him a to dentist, I show him the list of dentists in the city who have kindly remembered me and let him take his choice. My good friend Watson has told me I will have lots to answer for in extracting so many teeth. While I do extract a great many teeth, that it seems to me could be saved, yet, when a patient comes to me from a dentist with a card, having certain teeth marked upon it, I ask no questions but extract the teeth, unless it is evident that a mistake has been made in marking the wrong tooth, in which case I call up the dentist and make sure I am right before I go ahead. I am not supposed to know the history of the case with any tooth I am asked to extract for another dentist, nor do I consider it my business to find out. So if I am held accountable for these misdeeds, the other fellow will have to intercede for me and help me through.

If there is any one thing in the essayist's paper to which I would like to say *Amen* louder than to another, it is his caution against using new and untried remedies. In discussing this subject at the state meeting in July when the subject of Somnoforme was under discussion, I said that all such things should be experimented with in the colleges before the dentists at large used them; but when the Somnoforme people sent out reprints of Dr. Bowle's splendid paper, with the supposed discussions, they omitted enough of mine so that I was quoted as saying that "*dentists ought to experiment* with such things" which is entirely opposite to what I did say. We are too prone to pick up the use of new things which some times later cause us regret.

There is just one other thing I wish to speak about, although there are many other points brought out in the paper which deserve hearty support and this one no doubt the Dr. omitted, thinking it dwelt more upon our social life than it has to do with professional ethics. When I joined the society it was because I wanted to become a member and because I was invited to join. I believe there is a standing invitation now for any reputable ethical dentist in the city to become a member of our society. Then, each one of us is an invited guest and the society is the host. If you invite a guest into your home your very effort is bent upon pleasing him and any discourtesy to him is the farthest from your intentions. When I was a boy and anxious to do things that I thought would make me manly I tried hard to learn to smoke and the more I tried, the sicker I became until I gave up in despair; and it is only within the last few years that I was able to inhale much tobacco smoke without an actual attack of nausea. I have now, apparently become immune to the disagreeable effects and the only thing I have to contend with is the tendency toward domestic infelicity when I enter my home with my clothing and hair saturated with tobacco smoke and compel my good wife, to whom tobacco is especially obnoxious, to inhale the odor until time and the gentle breezes have done their work of fumigating. I have also two boys growing up whom I am trying to influence against being as foolish as their father was at their age, and when I come home in the evening after being in a smoke house, I am soon reminded that I have been to the club, the lodge or the dental society meeting. There is a guest of this society who has not yet passed through enough degrees to make him immune to the effects from tobacco smoke, and therefore absents himself from these meetings. Now he was invited to come, and then we make it so disagreeable for him that he cannot stay with us. Are you at liberty to pass it off lightly, or tell him if he does not like our style he can stay away? Or will you stamp him as a fool, a "sissy" or too effeminate? At the last meeting of this society Dr. Zederbaum brought his wife along with him, and I remember seeing only two or three cigars while she was in the hall. It reminded me very much of a story told of General Grant. When two or more of the sub-ordinate officers were alone with him at one time, one of them, desirous of relating a story, the propriety of which was in question, looked around the room and remarked: "There are no ladies present, are there?" When General Grant remarked: "No, but there are at least two or three gentlemen present."—The story was not told. I have often thought that it would be a good thing for some of us to bring our wives to our dental society meetings.

Dr. Shattuck: A preacher went out to take a new appointment and the trustees came to him and said: "Now, if you want to pitch into anybody, there is only one Jew in this town and he does not amount to anything, so you can pitch into him." Likewise I would say: There is only one college in this town, so our essayist pitches into it. I wonder what you gentlemen would do if you were running a college and had all these students with diverse traits of character to manage? I wonder how our essayist would do himself, if he had a college in charge, or even a certain portion of it. I wonder if would allow Tom, Dick and Harry through or whether he would pluck all of them? How would he find out when they cheated him? If any of you have had any experience with students, you know that they stand up for one another. They will lie for one another and they will steal from one another, and don't think anything about it. I know the colleges do not do as well as they ought to or would like to do. I know that men steal through the colleges, when the faculty is not looking at them, they slip through in spite of you. They come up to you with a good paper, they say: "I did this example without any help at all, and sign their name to it." What are you going to do about it? I would like to have some of these men that pitch into the colleges get into the college work. We want workers in our colleges. If any gentleman here thinks our college is not running right, come up there and I will give him a chance to take hold and show us. The college is struggling hard to build up the profession. I think that men who have a good deal to say against the colleges do not know what they have to go through as well as they would know if they were at the head of the college and woıking for it. Why don't these men get into the college and make one good and perfect college, one that is beyond suspicion? I do not mean this for Dr. Buttrick particularly, but there has been so much talk against the colleges. I have been teaching in colleges now twenty-four years, and in the dental college nearly fifteen, and in the medical colleges two or three of them, and I do not know as I ever heard anything about ethics in particular.

Dr. Wood: We are all to be congratulated on being able to listen to a paper from such a fearless man, one who lives up to every word of his creed as Dr. Buttrick. I think we have been "done brown" in ethics.

Dr. Nesbit: I have been pleased to listen to the paper of our worthy doctor. I have heard read a paper on the commercial side of the practice of dentistry, and here to-night this paper on the ethical side, and it seems to me the commercial and the ethical sides represent the two extremes of the swing of the pendulum, and if it be a judicious combination of the ethical and the commercial we get down to a practical basis. I think there are very few of us here who have not come into dentistry because of the necessity of some commercial basis, and the colleges which have prepared us are working out their salvation along the line of the commercial basis, and possibly the reason that we have not heard so much of the ethical side of the question, is that they wanted to be consistent. I am glad that this meeting represents a greater consistency along the line of ethics for you from my standpoint than many that I have observed.

When I graduated I was like one of the formeı speakers here, I joined this society, and I wanted to, and some friend I suppose presented my name. We met in a smaller room than this, and the air was none too good by reason of numbers but it was made worse by reason of the many smoking. To-night, I am pleased to say, this practice has been commendable from the view point of ethics. It is all right to talk about ethics in the office and relation of our treatment of our cases, and to the treatment of others in an ethical way, but why not let us be ethical all of the time if we can. Now I object to being smoked out. That is my good point—I do not smoke. I object to being smoked upon when I cannot get away. That is my privilege. I do not object to your smoking, but I do object to inhaling your sickening smoke myself. We come here first for business, the primary object of this society is to improve ourselves by the interchange of views and opinions, and if our conduct is such that it does not meet with the approval of our neighbor, is it not in our duty to get it into line with his views so that he can enjoy the meeting as we do ourselves? I am, therefore, glad that there has been an apparent improvement here to-night in that respect.

Now when the young men graduate from the college there are some of them on their elbows, or nearly so, and that person's first duty is to himself, and it is up to him to do the very best he can, and he cannot by reason of lack of funds be the ethical man that he would desire to be. Why is it that you can go into certain parts of this city and see such conditions as do not indicate that the people who live there have very extensive ideas along the line of ethics or ideals. They have not the means to make themselves idealistic or to work out their better conceptions. The commercial feature must and does form very largely the foundation of idealistic conceptions and ethics. All of us here have had certain privileges in the past conferred upon us, we have had the commercial feature—the dollar, so to speak—enough of it to give us a desire to go on to the ideal and to the ethical. In the early part of his practice the young dentist may not be as ethical as he would desire himself to be, and it is wise and ethical on the part of those who have got a standing by reason of graduating before this individual to be kind, considerate and not be too ready to blame him. He has a lot of things to learn; we all of us have a lot of things to learn, and I think the individual can largely solve the question of ethics for himself, by making one grand and good choice at some time of his life, whether it is before he graduates or after; and it has got to be the choice of doing right. If a man makes that choice for a life time, he has chosen righteousness, so to speak, as the motive principle. Then he has solved for himself the whole question of ethics very largely. It is possible that what is ethical for me may not be ethical for you. It is not a perpendicular line on which are arranged right on one side and wrong on the other; it is a variable line. Circumstances and environment, and a great many other factors enter into this thing, so that it behooves us to be kindly and considerate, and as ethical as we can be from our different view points.

DR. BUTTRICK: I did not imagine any of us had gone into dentistry from philantrophic motives. I know I did not. I believe if you work for a good fee you will work harder than if you are working for a nominal one. At the same time I believe in a square deal for all. Dr. Thompson spoke of it being rather of the nature of a theological discourse. I do not know that I should recognize the place from which some of those quotations come. He spoke about being able to belong to churches and societies and ride in automobiles and drive fast horses, and that sort of thing. My own view is that a man can belong to a church, or society, or drive fast horses, and be either ethical or unethical. I like very much the way Dr. Thompson put the commercial spirit as opposed to the professional one, the one was the joy of getting and the other the joy of giving. I can see quite a difference between what I term the business end of the profession and the commercial end of the profession. What I tried to say was that the unethical man is after his patient's dollars first; he does not care whether he does them any good or not, as long as he gets the money. The ethical man wants to do his patient as much good as he can.

A filling cannot do service unless it has proper contact with the approximating tooth and the opposing tooth as well, all of which should be brought to the patient's attention, and it should be left to him as a question of fee whether a failure in this regard should be left or repaired. Dr. Straith mentioned the fact that it might be a first bridge, and that you should handle it tenderly. I do not think it makes any difference whether it is the first or last bridge; the attention of the patient should be called to that bridge.

I did not speak of the question of discussing another's fees with the patient, as I do not think that is ethical. If you satisfy a patient that he wants good service, I think he will be willing to pay a good fee for it. My paper, as I said at the outset, was supposed to be impersonal; I was simply dealing with generalities. Dr. Shattuck was so kind as to say that he did not aim his remarks at me. I did aim a few of my remarks at the Detroit College, because it is my alma mater. If I am able to judge, there were a few ni our class who should not have been graduated. I do not believe in pitching on the only Jew in town either. I think our college is doing a good work, but there are some things that are not just as they should be, but that is the way with all colleges, and is also true of other things.

Dr. Nesbitt spoke in regard to the business end of the profession. I distinctly laid down the point that we should be well repaid for our services. We have taken a long and expensive course and it is not right to give away our services, but we should be ethical in doing our work and in collecting our fees.

